# Plasticity of TRPV1-Expressing Sensory Neurons Mediating Autonomic Dysreflexia Following Spinal Cord Injury

**DOI:** 10.3389/fphys.2012.00257

**Published:** 2012-07-09

**Authors:** Leanne M. Ramer, A. Peter van Stolk, Jessica A. Inskip, Matt S. Ramer, Andrei V. Krassioukov

**Affiliations:** ^1^International Collaboration On Repair Discoveries, University of British ColumbiaVancouver, BC, Canada; ^2^Faculty of Medicine, University of British ColumbiaVancouver, BC, Canada; ^3^Faculty of Medicine, Simon Fraser UniversityVancouver, BC, Canada; ^4^Department of Biomedical Physiology and Kinesiology, Simon Fraser UniversityVancouver, BC, Canada; ^5^Spinal Cord Injury Program, GF Strong Rehabilitation CentreVancouver, BC, Canada; ^6^Department of Medicine, University of British ColumbiaVancouver, BC, Canada

**Keywords:** capsaicin, colo-rectal distension, dorsal horn, dorsal root, dorsal root ganglion, high blood pressure, hypertension, hypertrophy

## Abstract

Spinal cord injury (SCI) triggers profound changes in visceral and somatic targets of sensory neurons below the level of injury. Despite this, little is known about the influence of injury to the spinal cord on sensory ganglia. One of the defining characteristics of sensory neurons is the size of their cell body: for example, nociceptors are smaller in size than mechanoreceptors or proprioceptors. In these experiments, we first used a comprehensive immunohistochemical approach to characterize the size distribution of sensory neurons after high- and low-thoracic SCI. Male Wistar rats (300 g) received a spinal cord transection (T3 or T10) or sham-injury. At 30 days post-injury, dorsal root ganglia (DRGs) and spinal cords were harvested and analyzed immunohistochemically. In a wide survey of primary afferents, only those expressing the capsaicin receptor (TRPV1) exhibited somal hypertrophy after T3 SCI. Hypertrophy only occurred caudal to SCI and was pronounced in ganglia far distal to SCI (i.e., in L4-S1 DRGs). Injury-induced hypertrophy was accompanied by a small expansion of central territory in the lumbar spinal dorsal horn and by evidence of TRPV1 upregulation. Importantly, hypertrophy of TRPV1-positive neurons was modest after T10 SCI. Given the specific effects of T3 SCI on TRPV1-positive afferents, we hypothesized that these afferents contribute to autonomic dysreflexia (AD). Rats with T3 SCI received vehicle or capsaicin *via* intrathecal injection at 2 or 28 days post-SCI; at 30 days, AD was assessed by recording intra-arterial blood pressure during colo-rectal distension (CRD). In both groups of capsaicin-treated animals, the severity of AD was dramatically reduced. While AD is multi-factorial in origin, TRPV1-positive afferents are clearly involved in AD elicited by CRD. These findings implicate TRPV1-positive afferents in the initiation of AD and suggest that TRPV1 may be a therapeutic target for amelioration or prevention of AD after high SCI.

## Introduction

The original formulation of the neurotrophic hypothesis by Hamburger and Levi-Montalcini (1949) asserted that the survival of neurons depends on cues produced in limiting amounts in target tissues. The subsequent discovery of nerve growth factor (NGF) as a survival factor for sympathetic and sensory neurons (Cohen et al., 1954) not only validated this hypothesis, but also extended the role of neurotrophic factors beyond developmental neuronal survival: target-derived NGF and numerous other neurotrophic molecules maintain the phenotype of neurons during adulthood. This has been arguably best demonstrated in experiments on primary afferent neurons of the dorsal root ganglion (DRG), a heterogeneous population of neurons responsible for transmission of information from the periphery (somatic) and internal milieu (visceral) to the spinal cord and brainstem.

Early classification of DRG neuronal subsets was based on cytoplasmic staining with standard histological techniques and electrophysiological recording followed by dye-filling of neurons (Willis and Coggeshall, 2004). These methods revealed “small dark” neurons which have slowly conducting axons (corresponding to thermoceptors and nociceptors, approximately 70% of all neurons in the DRG), and “large light” cells with rapidly conducting fibers (30% of all DRG neurons, representing skin and muscle mechanoreceptors). In terms of their neurochemistry, DRG neurons are remarkably diverse, and much effort has gone into establishing particular neurochemical phenotypes as functional proxies (Willis and Coggeshall, 2004). Among the small dark cells, for example, there are peptidergic and non-peptidergic subtypes, which also happen to be sensitive to different neurotrophic factors (NGF, and glial cell line-derived neurotrophic factor, GDNF, respectively; Braz et al., 2005). Ascribing functional relevance to this neurochemical distinction has proven difficult, in part because both populations express transducers of thermal and noxious stimuli: one example is the capsaicin-sensitive cation channel transient receptor potential vanilloid receptor (TRPV1), a remarkably versatile receptor that is also activated by protons and noxious heat (Caterina et al., 1997; Tominaga et al., 1998).

Dorsal root ganglia neurons undergo marked changes in neuronal phenotype following their disconnection from their target tissues by axotomy. Importantly, and in-line with the neurotrophic hypothesis, these changes are mimicked by depletion of the relevant factors in the absence of axotomy, and reversed by supplying axotomized neurons with trophic support from exogenous sources (Rich et al., 1984; Yip et al., 1984; Johnson and Yip, 1985; Wong and Oblinger, 1991; Matheson et al., 1997; Bennett et al., 1998). Uninjured DRG neurons undergo hypertrophy following *in vivo* delivery of NGF or GDNF (Ramer et al., 2001, 2003). Likewise, transgenic over-expression of NGF (Goodness et al., 1997) and the GDNF family member artemin (Elitt et al., 2006) cause sensory neuronal hypertrophy.

Spinal cord injury (SCI), while not disconnecting DRG neurons from their peripheral targets, nevertheless has profound effects on multiple tissues which might be expected to influence neuronal phenotype. Among the most dramatic of these are skeletal muscle atrophy, and in the lower urinary tract (LUT), detrusor hypertrophy. In the latter case, increased NGF production by the bladder has been correlated with hypertrophy of innervating DRG neurons (Yoshimura, 1999). For complete lesions above T5/6, SCI is almost always accompanied by cardiovascular disturbances including orthostatic hypotension (OH; a sudden fall in blood pressure upon assuming an upright position) and autonomic dysreflexia (AD; potentially life-threatening elevations in blood pressure triggered by sensory stimulation below the injury; Krassioukov and Claydon, 2006). There is increasing evidence that blood vessels, a peripheral target of sensory as well as sympathetic axons, also undergo SCI-induced alterations which may evoke phenotypic changes in their innervating neurons (McLachlan and Brock, 2006; Alan et al., 2010; Rummery et al., 2010; Al Dera et al., 2011; Tripovic et al., 2011).

Here we took a systematic immunohistochemical approach to studying one of the defining phenotypic features of sensory neurons – soma size – in different populations of sensory neurons after T3 SCI. We characterized hypertrophy in a specific subset of nociceptors, those that are sensitive to capsaicin and artemin. We examined the effect of rostro-caudal level and injury level; we also examined the density of central projections of hypertrophied neurons and the role of those neurons in AD. We hypothesized that SCI would induce nociceptor hypertrophy and/or terminal sprouting and that these effects would be more pronounced after high-thoracic injury. After identifying a specific subset of sensory neurons that responded to SCI, we hypothesized that these would be involved in induction and/or development of AD.

## Materials and Methods

### Spinal cord injury surgery

Complete transection of the spinal cord at the third (T3) or tenth (T10) thoracic segments was performed in adult male Wistar rats (250–350 g, Charles River Laboratories, Inc., Wilmington, Canada). T3 SCI promotes the development of cardiovascular dysfunction, including AD, while T10 SCI does not. Sham surgeries were performed at T3 and were identical up to and including durotomy, without transection of the cord.

Rats were housed in a secure conventional rodent facility, on a 12-h reversed light-dark cycle. The surgical procedures and post-operative animal care have been described in detail (Ramsey et al., 2010), but the essential steps are included here.

Animals received prophylactic enrofloxacin (Baytril; 10 mg/kg, s.c., Associated Veterinary Purchasing; AVP, Langley, Canada) for 3 days prior to SCI surgery. On the day of surgery, anesthesia was induced with ketamine hydrochloride (Vetalar^®^; 70 mg/kg, i.p., University of McGill Animal Resources Centre, Montreal, Canada) and medetomidine hydrochloride (Domitor^®^; 0.5 mg/kg, i.p., AVP). Enrofloxacin (10 mg/kg, s.c.), buprenorphine (Temgesic^®^; 0.02 mg/kg, s.c., McGill University), and ketoprofen (Anafen^®^, 5 mg/kg, s.c., AVP) were administered pre-operatively.

After the skin at the surgical site was shaved, scrubbed, and treated with iodine, the animal was placed in the prone position. The spinal cord was exposed *via* a midline incision in the skin and superficial muscles, and blunt dissection of the muscles overlying the C8–T3 (T3 transection) or T8–T11 (T10 transection) vertebrae. At the T2–T3 intervertebral space or following a small laminectomy at T9, connective tissues were removed, the dura was opened and the spinal cord was completely transected with surgical scissors. Complete transection was verified by the clear separation and retraction of the cut ends of the cord, visualized under the surgical microscope. After hemostasis was achieved, the muscle and skin were closed with continuous, 4-0 Vicryl sutures, and interrupted 4-0 Prolene sutures, respectively.

### Post-operative animal care

Animals received warmed Lactated Ringer’s solution (5 ml, s.c.) and recovered in a temperature-controlled environment (Animal Intensive Care Unit, HotSpot for Birds, Los Angeles, CA, USA). Anesthesia was reversed with atipamezole hydrochloride (Antisedan; 1 mg/kg, s.c., Novartis, Mississauga, Canada). Enrofloxacin (10 mg/kg, s.c.), buprenorphine (0.02 mg/kg, s.c.), and ketoprofen (5 mg/kg, s.c.) were administered once per day for 3 days following SCI.

Home cages for animals with complete T3 SCI were fitted with rubber matting (under woodchips, to facilitate movement), low-reaching water bottles, and food scattered on the cage bottom to encourage foraging (Ramsey et al., 2010). Animals were supported with an enriched diet, including meal replacement shake (Ensure; Abbott, Saint-Laurent, Canada), nutritive transport gel (Charles River), fruit, cereals, commercially available rat treats and kibble (LabDiet, Rodent Diet 5001). The bladder was manually expressed three to four times daily until spontaneous bladder function returned (about 10 days post-injury). Animal health was formally monitored daily for the first 2 weeks after SCI and every 2 days thereafter, using objective criteria to assess body weight, activity level, social behavior, healing at the surgery site, and clinical signs of morbidity.

### Survival times

Animals survived for 1 or 3 months after SCI. In our initial experiments examining SCI-induced hypertrophy in the DRG (data in Figures 1–3 and 5), T3 sham-, and spinal cord-injured animals survived for 3 months. In all subsequent experiments (data in Figures 4 and 6–8), T3 and T10 sham and spinal cord-injured animals survived for 1 month following surgery.

**Figure 1:**
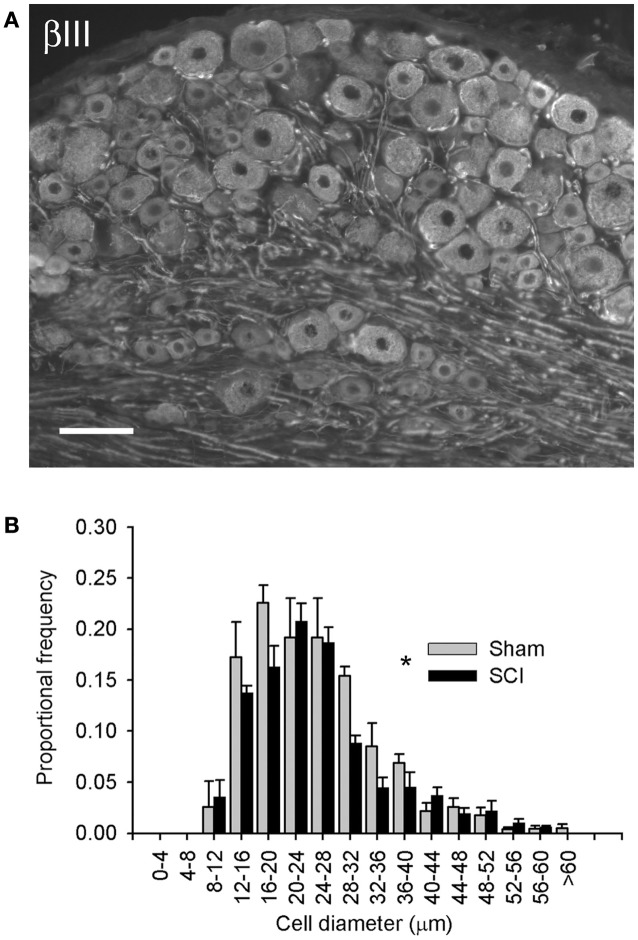
**High-thoracic (T3) spinal cord injury provoked hypertrophy of neurons in the L4/L5 dorsal root ganglion**. **(A)** βIII-tubulin immunohistochemistry illustrating neuronal profiles in the L5 DRG. **(B)** Size-frequency distribution of pooled L4/L5 DRG neurons, reconstructed from profile distributions using recursive translation. Ganglia were harvested 3 months after sham-injury (gray) or complete T3 SCI (black). There was a small but significant rightward shift in size-frequency distribution in animals with T3 SCI (*P* < 0.05, K–S goodness-of-fit test). Scale bar = 70 μm.

**Figure 2:**
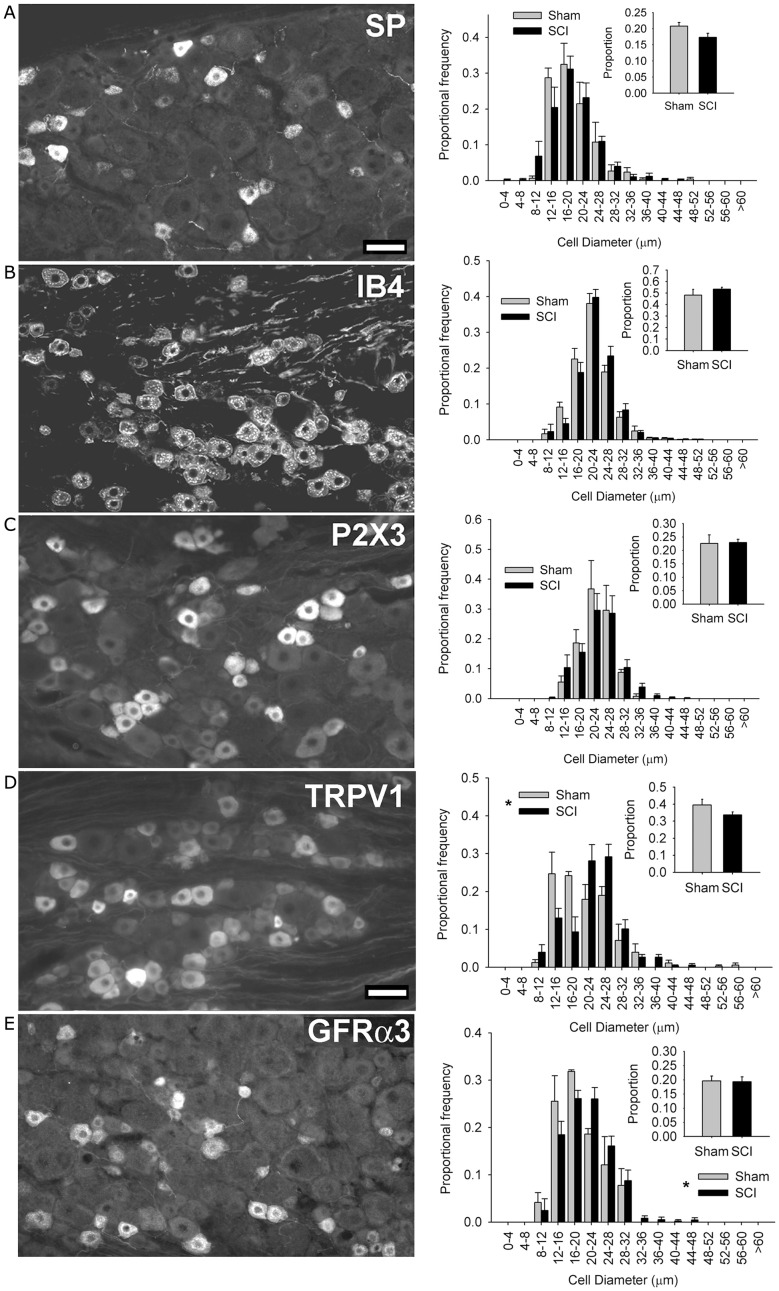
**High-thoracic (T3) spinal cord injury-induced selective hypertrophy of sensory neurons expressing the capsaicin receptor (TRPV1) and the artemin receptor (GFRα3) in the L4/L5 DRG**. **(A)** Substance P (SP) – positive DRG neurons and their size-frequency distributions. **(B)** IB4-binding DRG neurons. **(C)** P2X3-positive DRG neurons. **(D)** TRPV1-positive DRG neurons. **(E)** GFRα3-expressing DRG neurons, known to express TRPV1. The overall proportions of immunopositive neurons [insets **(A–E)**] did not change for any subpopulation. Ganglia were harvested 3 months after sham-injury (gray) or complete T3 SCI (black). Scale bar = 50 μm. Asterisks indicate *P* < 0.05, K–S goodness-of-fit test.

**Figure 3:**
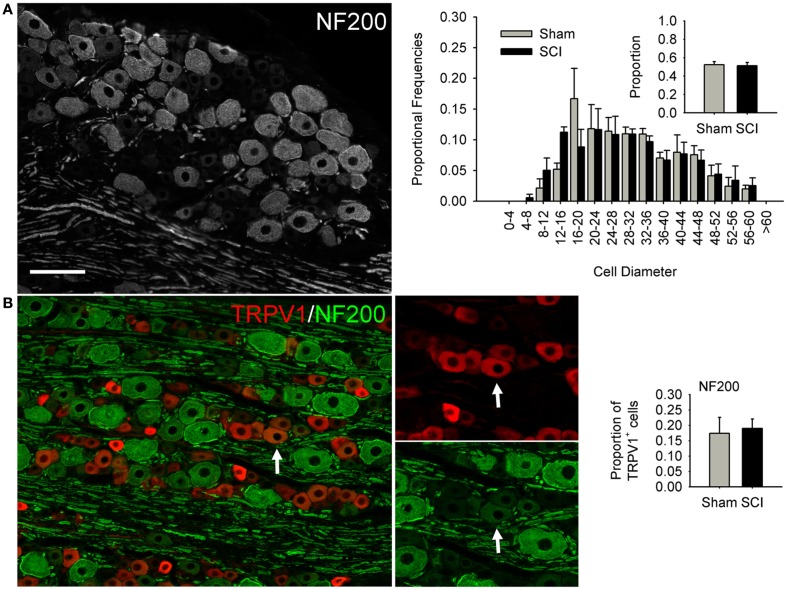
**High-thoracic (T3) spinal cord injury had no effect on medium-to-large sized neurons in the L4/L5 DRG expressing heavy neurofilament (NF200)**. **(A)** NF200-positive neurons did not undergo SCI-induced hypertrophy, nor did the proportion of neurons expressing NF200 change. **(B)** Hypertrophy of TRPV1-expressing DRG neurons was not accompanied by increased co-localization of TRPV1 and NF200. Ganglia were harvested 3 months after sham-injury (gray) or complete T3 SCI (black). Arrow: DRG neuron immunopositive for both TRPV1 and NF200. Scale bar = 70 μm.

**Figure 4:**
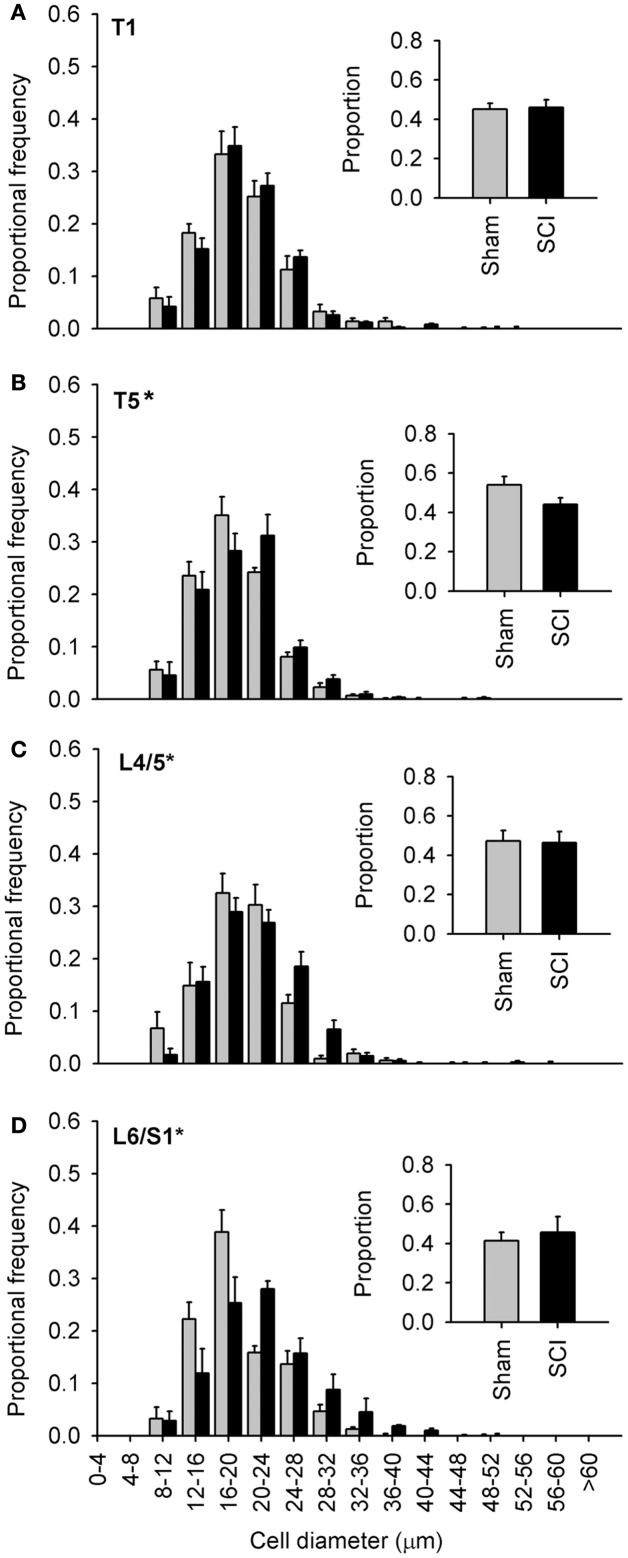
**Capsaicin-sensitive dorsal root ganglion neurons increased in diameter caudal to, but not rostral to, high-thoracic spinal cord injury**. **(A–D)** Size-frequency distributions of TRPV1-positive neurons from a rostral DRG (T1) and caudal (T5, L4/L5, L6/S1) DRGs, harvested 1 month after sham- (gray) or complete T3 SCI (black). The increase in size is the most pronounced in the most caudal ganglia. Proportions of TRPV1-positive neurons at each level are shown in the insets **(A–D)**. Asterisks indicate *P* < 0.05, K–S goodness-of-fit test.

**Figure 5:**
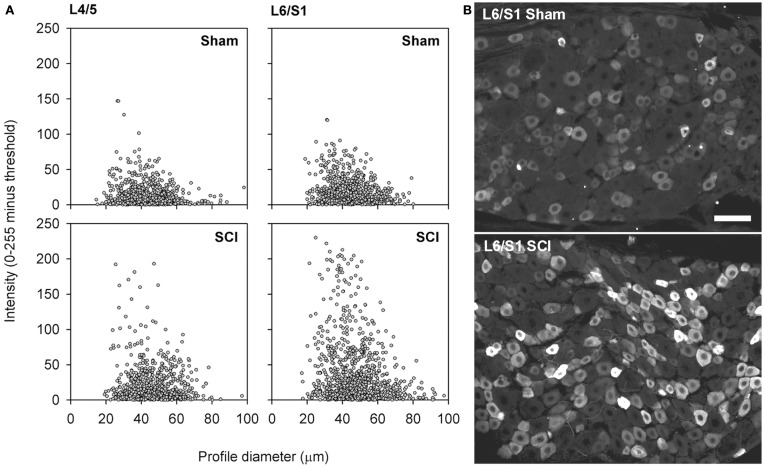
**Capsaicin-sensitive neurons in the dorsal root ganglion exhibited increased TRPV1 signal intensity following high-thoracic spinal cord injury**. **(A)** Size-intensity scatter plots of TRPV1-positive DRG neurons showing a marked upward scatter with T3 SCI. The increase in TRPV1 intensity was particularly pronounced in L6/S1 DRGs. There was a significant increase in signal intensity at 3 months after T3 SCI for both L4/L5 DRGs and L6/S1 DRGs (K–S goodness-of-fit tests on cumulative intensity-frequency distributions). **(B)** Representative images of TRPV1-expression in the L6/S1 DRG, from a sham-injured control (top) and an animal with T3 SCI (bottom). Scale bar = 50 μm.

**Figure 6:**
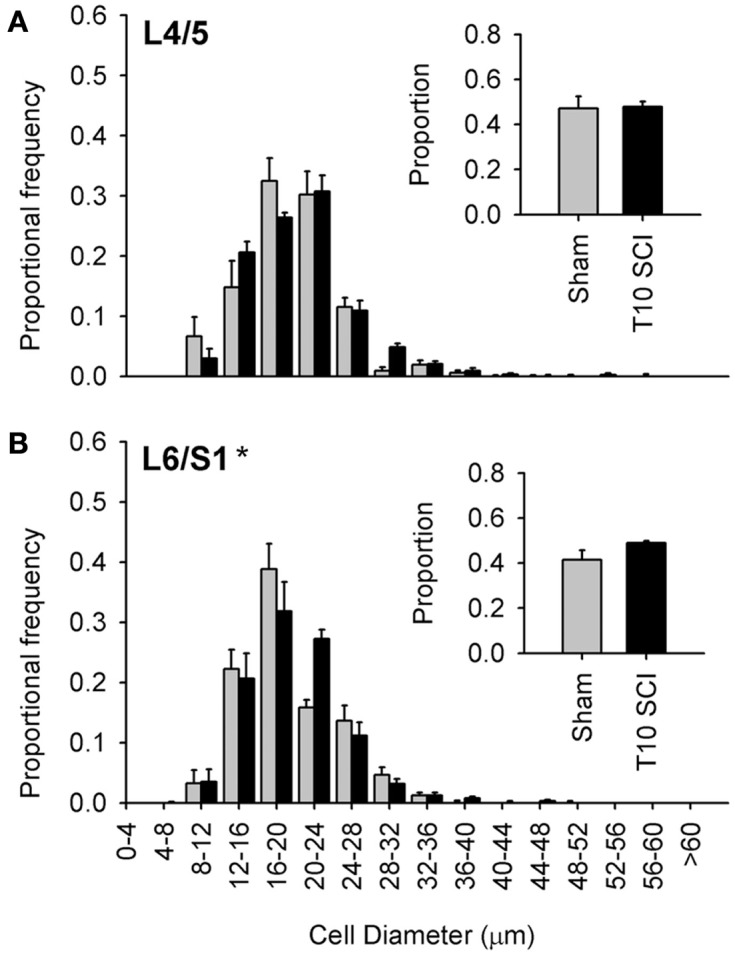
**Low-thoracic (T10) spinal cord injury elicited only modest changes in size of the most caudal capsaicin-sensitive dorsal root ganglion neurons**. **(A)** No difference in size distributions of TRPV1-expressing DRG neurons in L4/L5 DRGs. **(B)** There was a small but significant rightward shift in the size-frequency distribution of L6/S1 DRG neurons (*P* < 0.05, K–S goodness-of-fit test), but this was much less dramatic than that which occurred after T3 SCI (see Figure 4). Ganglia were harvested 1 month after sham (gray) or complete T3 SCI (black).

**Figure 7:**
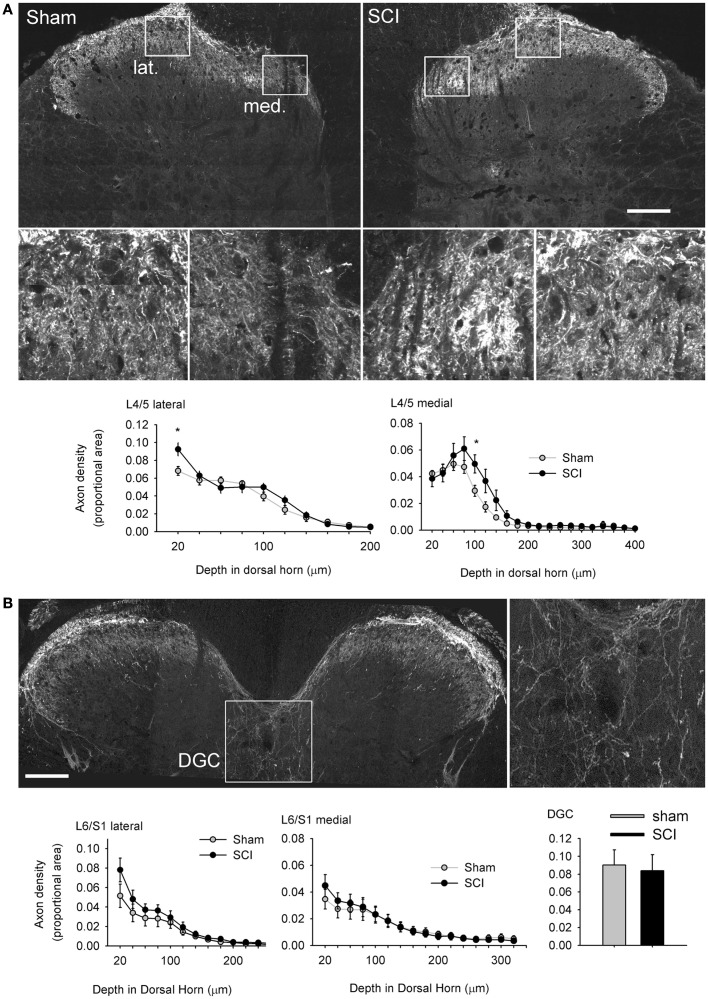
**Hypertrophy of capsaicin-sensitive afferents caudal to high-thoracic spinal cord injury was not accompanied by pronounced plasticity of their spinal projections**. **(A)** One month after T3 complete SCI, there was a small but significant increase in TRPV1-positive axon density in the medial (med.) and lateral (lat.) parts of the most superficial laminae of the L4/L5 dorsal horn (boxed regions are shown enlarged). There was no evidence of TRPV1-positive axon extension into deeper laminae. **(B)** In the L6/S1 cord, there were no differences between sham-injured or T3 SCI animals in the dorsal horn, or in the dorsal gray commissure (DGC, boxed image enlarged to the right), the terminal field of the visceral medial collateral pathway. Asterisks: *P* < 0.05, Student’s *t*-test. Scale bars = 200 μm.

**Figure 8:**
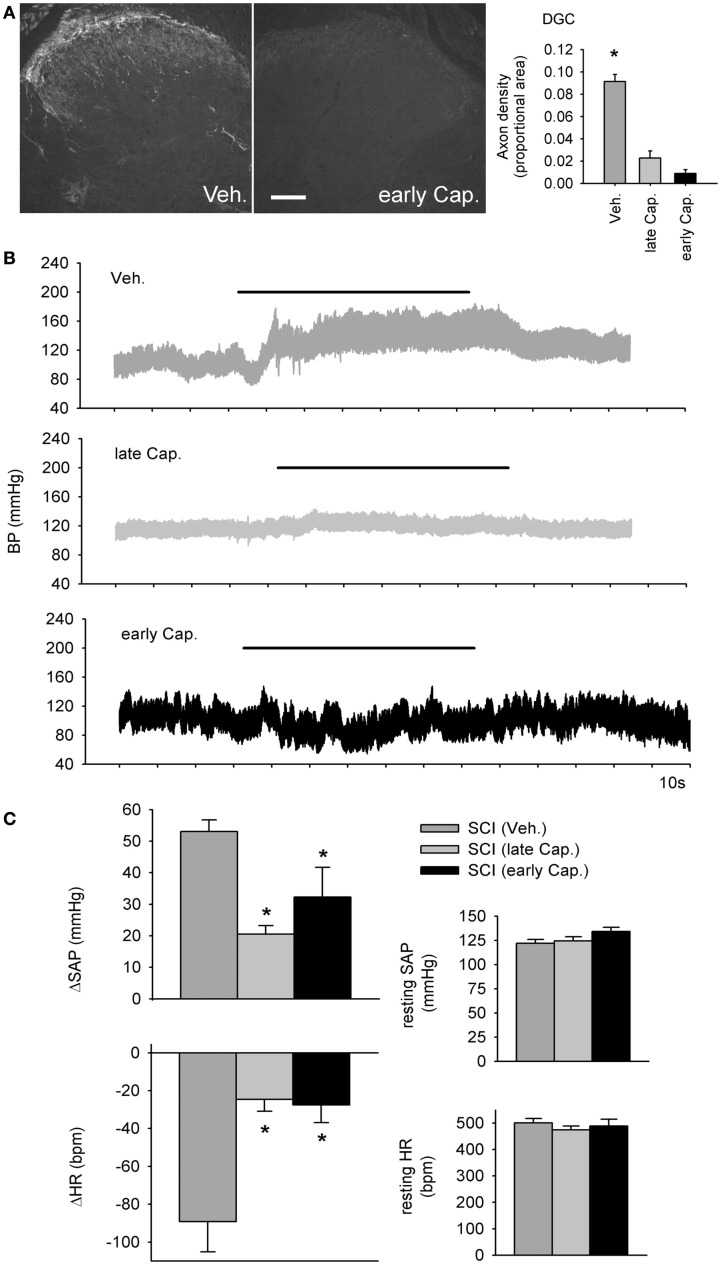
**Intrathecal capsaicin attenuated colo-rectal distention (CRD) -induced autonomic dysreflexia in animals that survived for 1 month after complete T3 spinal cord injury**. **(A)** A single intrathecal bolus of capsaicin (10 μl of 5 mg/ml in 50% DMSO) resulted in permanent degeneration of spinally projecting TRPV1-positive axons. Quantification shows TRPV1 axon density in the dorsal gray commissure (DGC). Asterisk indicates significant difference between vehicle-treated T3 SCI animals and both capsaicin-treated groups (*P* < 0.05, one-way ANOVA). Scale bar = 200 μm. **(B)** Beat-to-beat changes in blood pressure in response to colo-rectal distension (horizontal bars) from animals treated with intrathecal vehicle (Veh.), early capsaicin (48 h following T3 SCI, early Cap.), and late capsaicin (48 h prior to physiological recording, 28 days post-T3 SCI, late Cap.). **(C)** Quantitative cardiovascular responses to CRD in vehicle and capsaicin-treated rats, 30 days post-SCI. Capsaicin treatment, whether administered 48 h or 28 days after SCI, mitigated CRD-induced increases in systemic arterial pressure (SAP) and decreases in heart rate (HR). Resting SAP and HR were unaffected by intrathecal capsaicin. Asterisks: *P* < 0.05, one-way ANOVA.

### Intrathecal capsaicin injection

At 48 h (early cap.) or 28 days (late cap.) after SCI, animals were anesthetized with inhalant isoflurane (AErrane^®^, AVP; 5% induction, 2–3% maintenance). The skin overlying the lumbar enlargement of the spinal cord was shaved, scrubbed, and treated with iodine. The lumbar enlargement was exposed *via* midline incision in the skin and superficial muscles, blunt dissection of deeper muscles, and a midline laminectomy. Polyurethane tubing (PU, 3 French, Instech, Plymouth Meeting, USA) was introduced sub-durally and 50 μg of capsaicin (Sigma-Aldrich Inc., St. Louis, USA) in 10 μl 50% dimethyl sulfoxide (DMSO; Sigma) was injected intrathecally. Control animals received 50% DMSO only, at 28 days after SCI. The muscle and skin were closed with sutures (4-0 Vicryl and 4-0 Prolene, respectively).

### Cardiovascular assessment

Thirty days after SCI, a cannula (PU, 3 French, Instech) was implanted into the left carotid artery in all animals for continuous beat-to-beat blood pressure recording. Arterial cannulation was performed under isoflurane anesthesia and the cannula was tunneled subcutaneously to exit the skin dorsally, through small incision at the base of the skull. The cannula was filled with a lock solution of 1:10 heparin (Hepalean^®^, AVP) and 5% dextrose in Lactated Ringer’s.

Two hours after carotid cannulation, the cannula was connected to a fluid-filled pressure transducer (SP844, MEMScAP, Norway). Animals were conscious and the cannula was long enough to permit them to move freely in a cage during cardiovascular assessment. Beat-to-beat arterial pressure was monitored using PowerLab and Chart™ 5 for Windows (ADInstruments, Colorado Springs, USA). When blood pressure was stable (typically 5–10 min after connecting the cannula to the transducer), baseline blood pressure was recorded over 5 min.

The severity of colo-rectal distension (CRD)-induced AD was examined using a protocol that is well-established in our laboratory (Krassioukov and Weaver, 1995). A pediatric silicone balloon-tipped catheter (10 French; Coloplast, Denmark) was inserted into the rectum and secured to the tail. After stabilization of arterial pressure, the colon was distended *via* inflation of 2 ml of air, over 10 s. Distension was maintained for 1 min; upon deflation of the balloon, arterial pressure was allowed to recover over 10 min. Blood pressure was recorded during two episodes of AD in each animal, with a minimum of 10 min of recovery intervening.

### Tissue processing and immunohistochemistry

Animals were euthanized with an overdose of chloral hydrate (1 g/kg, i.p.) and perfused through the heart with room temperature phosphate-buffered saline (PBS) followed by cold 4% paraformaldehyde (PF). Spinal segments and DRGs were removed, post-fixed in 4% PF for 12 h and cryoprotected in 20% sucrose in 0.1 M phosphate buffer for ≥24 h. Tissue was embedded in Tissue Tek (Fisher Scientific, Ottawa, Canada), frozen over liquid nitrogen, sectioned on a cryostat at 16 μm (DRG) or 20 μm (cord), thaw-mounted on to glass slides and stored at −80°C.

For immunohistochemistry, slides were incubated in 10% normal donkey serum in PBS plus Triton X-100 (0.1%) for 20 min. We used five antibodies to delineate different subsets of nociceptors: these included antibodies raised against TRPV1 (Neuromics, Edina, MN, USA; 1:2,000), the ionotropic ATP purinoceptor P2X_3_ (Millipore, Billerica, MA, USA; 1:1,000), Substance P (Neuromics, 1:1,000), the glycoprotein isolectin B4 (IB4; Neuromics; 1:2,000), the glial cell line-derived neurotrophic factor family receptor α 3 (GFRα3; R&D Systems; 1:500). Subsets of non-nociceptive sensory neurons were identified by expression of the vanilloid transient receptor potential vanilloid-2 (TRPV2; Abcam, Cambridge, UK; 1:200), parvalbumin (Millipore, Etobicoke, Canada; 1:1,000), stage-specific embryonic antigen-4 (SSEA-4; Stem Cell Technologies, Vancouver, Canada; 1:100), and heavy neurofilament (NF200; Millipore, 1:500). Pan-neuronal markers – microtubule-associated protein-2 (MAP-2; Abcam, 1:5:000) or β-III-tubulin (Neuromics; 1:500) – were used to label all neuronal profiles in the DRG.

All primary antibodies were applied in PBS plus Triton X overnight. After three 15-min washes in PBS, secondary antibodies raised in donkey and conjugated to Cy3 (Jackson ImmunoResearch, West Grove, USA), Alexa 488 (Invitrogen, Eugene, USA), or AMCA (7-amino-4-methylcoumarin-3-acetic acid; Jackson ImmunoResearch) were applied at 1:100–1:400, in PBS-Triton X for 2 h. Epifluorescent images of DRGs were captured with an Axioplan 2 microscope (Zeiss, Jena, Germany) with a digital camera (Q Imaging, Burnaby, Canada) and Northern Eclipse software (Empix Imaging, Inc., Mississauga, Canada). Confocal images of TRPV1-positive axons in the spinal cord were captured on a spinning disk confocal microscope (an inverted Zeiss AxioObserver Z.1 equipped with an AxioCam CCD camera). All images for each antigen used for quantitative analysis were captured at identical imaging settings.

### Cardiovascular data analysis

Systolic and diastolic blood pressures (SAP and DAP, mmHg) were obtained from maxima (max) and minima (min) respectively of beat-to-beat blood pressure recordings. Mean arterial pressure (MAP) was calculated as 1/3max + 2/3min and heart rate (HR, beats per minute, bpm) was calculated from inter-beat interval. Prior to data analysis, raw beat-to-beat blood pressure data were examined in the Chart view; false max/min readings created by muscle spasm were manually eliminated for each animal. Data were analyzed using SigmaPlot (SPSS Inc., Ashburn, USA). In SigmaPlot, raw pressure and HR values were averaged over 1 s, such that measurements represent a 1-s average, not a single beat. For each animal, baseline cardiovascular parameters represent the average of at least 3 min of recording time. The CRD-evoked changes in SAP and HR represent the average of two consecutive distensions for each animal.

### Image analysis

All images were analyzed using SigmaScan Pro 5.0 (SPSS Inc.). For analysis, L4 and L5 DRGs were pooled for each animal (denoted as L4/L5 throughout) and L6 and S1 DRGs were pooled for each animal (L6/S1 throughout). The size-frequency distributions of sensory neurons in the DRG were determined using recursive translation (Rose and Rohrlich, 1988), which converts neuronal profiles in section to the cellular population from which they were drawn (as described previously; Ramer et al., 2001). In five randomly selected sections from each DRG, all neuronal profiles were traced manually to create an artificial overlay. The average intensity across the cell body and soma diameter of each neuronal profile identified by the overlay was measured automatically; soma diameter was defined as Feret diameter, the theoretical diameter of the object if it were circular in shape. For proportional frequency measurements, the threshold intensity for expression was set manually for each image. Size-frequency and intensity-frequency distributions were generated to examine SCI-induced shifts in afferent size and TRPV1 signal intensity, respectively.

In the spinal cord, measurements of the distribution and intensity of TRPV1-positive axons in the dorsal horn were taken from tiled confocal projected z-stacks of the L4/L5 and L6/S1 dorsal horn in cross section. Terminal density was measured as a function of depth at two sites in the dorsal horn (mid and medial) as described previously (Ramer et al., 2001, 2004; MacDermid et al., 2004; Scott et al., 2005). Images were passed through a Laplacian omnidirectional edge-detection filter, which optimizes the signal-to-noise ratio and corrects for variations in background staining. Terminal profiles in filtered images were selected with a threshold overlay. In order to give all immunopositive pixels equal weight regardless of brightness, the threshold overlay was treated as a new image. Density measurements for each animal represent average density at each depth across five sections.

Density of TRPV1-positive axons in the spinal parasympathetic nucleus was measured from confocal images of the L6/S1 dorsal gray commissure (DGC). These images were filtered through horizontal and vertical Sobel edge-detection filters, with the results added through image math prior to thresholding. Density was measured in the spinal parasympathetic nucleus by selecting an area of gray matter that was centered on the midline, immediately ventral to the dorsal corticospinal tract and rostral to the central canal. Density measurements for each animal represent average density across three to five sections.

### Statistics

For cardiovascular data, baseline parameters and stimulus-evoked changes were compared among groups using a one-way analysis of variance (ANOVA). The Holm–Sidak Test was used for pair-wise comparisons when significant differences were detected. For size distribution data from the DRG, Kolmogorov–Smirnov (K–S) goodness-of-fit tests were used to determine whether neuronal cumulative size-frequency distributions differed between groups (sham- versus spinal cord-injured). Proportions of neurons labeled with each antigen were compared using Student’s *t*-test. The K–S goodness-of-fit test was also used to detect inter-group differences in cumulative intensity-frequency distributions of TRPV1-expressing cells in the DRG. Density of TRPV1-positive terminals in the dorsal horn of sham- and spinal cord-injured animals was compared using a one-way ANOVA on ranks, followed by Dunn’s test to detect pair-wise differences. Density of TRPV1-positive terminals in the DGC of sham-injured and SCI rats was compared between groups using Student’s *t*-test. In assessing the effects of early and late intrathecal capsaicin on TRPV1 axons in the DGC, a one-way ANOVA was used. For all physiological and anatomical analyses, group averages represent five to seven animals per group, results are expressed as mean ± standard error of the mean (SEM), image, and data analyses were performed in a blinded fashion (using coded image and data files) and *P* values less than 0.05 were considered significant.

## Results

### Complete high-thoracic spinal cord injury provoked hypertrophy in sensory neurons that express the capsaicin receptor

Neuronal phenotype, of which size is a defining characteristic, is governed in large part by trophic influences of target tissues. These undergo profound changes following SCI, including atrophy of skeletal muscle and bladder hypertrophy. Therefore, we examined changes in the size distribution of all sensory neurons in lumbosacral DRGs 3 months after T3 complete SCI. Neuronal profiles in the L4/L5 DRG were labeled with neuron-specific β-III-tubulin (Figure 1A) and size-frequency analysis was performed following recursive translation (Figure 1B). There was a subtle but statistically significant right-shift (i.e., hypertrophy) in the size distribution of all sensory neurons in DRGs of animals with T3 SCI.

We next sought to determine which population(s) of sensory neurons responded to T3 SCI. We began by examining subsets of small-diameter sensory neurons in the L4/L5 DRG (Figure 2). Peptidergic (NGF-sensitive) nociceptors were identified by expression of Substance P (SP; Figure 2A), while non-peptidergic nociceptors were identified by binding of the glycoprotein isolectin B4 (IB4, from *Bandeiraea simplicifolia*) and the ionotropic ATP purinoceptor P2X_3_ (Figures 2B,C). At 3 months after T3 SCI, neither of these minimally overlapping populations of nociceptors exhibited hypertrophy after T3 SCI, suggesting that sensory hypertrophy caudal to T3 SCI was selective to another subset of sensory neurons.

In rats, TRPV1 expression occurs in subsets of both peptidergic and non-peptidergic nociceptors (Tominaga et al., 1998). TRPV1 expression also defines a subpopulation of neurons which are neither P2X_3_-expressing/IB4-binding nor neuropeptide-expressing (Michael and Priestley, 1999). When we examined the size distribution of TRPV1-positive neurons in the L4/L5 DRG, we found a pronounced hypertrophy in DRGs from animals with T3 SCI (Figure 2D). TRPV1-positive cells increased in size after SCI, but their proportional frequency did not change (Figure 2D, inset). Approximately 50% of TRPV1-positive sensory neurons also express the artemin-specific glial cell line-derived neurotrophic factor family member GFRα3 (Baloh et al., 1998; Bennett et al., 2006). GFRα3-positive sensory neurons (of which >80% express TRPV1; Bennett et al., 2006) also exhibited hypertrophy, in the absence of change in proportional frequency, in DRGs distal to T3 SCI (Figure 2E).

We performed size-frequency analysis on medium-to-large sensory neurons in the same (L4/L5) DRGs by examining neurons expressing heavy neurofilament (NF200; Figure 3). At 3 months after T3 SCI, there was no evidence of injury-induced hypertrophy in NF200-positive cells, nor did the proportion of NF200-expressing cells change (Figure 3A). In agreement with previous findings (e.g., Yamamoto et al., 2008), we found that the large majority of TRPV1-positive neurons were NF200-negative; only occasional neurons co-expressed TRVP1 and NF200 (Figure 3B, arrow). The extent of TRPV1 and NF200 co-expression did not increase after T3 SCI (Figure 3B).

To confirm that SCI-induced hypertrophy was specific to small-diameter DRGs, we also performed size-frequency analysis on three subpopulations of medium-to-large sensory neurons in the L4/L5 DRG (data not shown). Expression of TRPV2 was used to identify larger sensory neurons that are heat-sensitive but TRPV1-negative (Caterina et al., 1999; Tamura et al., 2005). Proprioceptors and cutaneous mechanoreceptors were labeled with parvalbumin (Celio, 1990) and SSEA-4 (Dodd et al., 1984), respectively. In contrast to SCI-induced hypertrophy in TRPV1-expressing nociceptors, there were no detectable changes in the size distributions of these subpopulations of medium- to-large DRG neurons following T3 SCI.

### Spinal cord injury-induced hypertrophy was most pronounced in lumbosacral sensory ganglia

Since our initial observations were made in DRGs far distal to T3 SCI (Figures 1–3, L4/L5 DRG), we examined the extent of SCI-induced hypertrophy in TRPV1-positive neurons at different rostro-caudal levels (Figure 4). We examined tissue harvested 1 month after T3 durotomy (sham-injury) or SCI (Figures 4–6). Of the levels examined, SCI-induced hypertrophy was restricted to ganglia below the injury: TRPV1-positive afferents did not exhibit hypertrophy in the T1 DRG, despite their proximity to the injury site (Figure 4A). Equidistant but caudal to SCI (in the T5 DRG), the size distribution of TRPV1-positive sensory neurons was right-shifted relative to sham-injured controls (Figure 4B). However, the effect of SCI was most dramatic in lumbosacral DRGs, remote from the site of injury. TRPV1-positive cells exhibited pronounced hypertrophy in both L4/L5 and L6/S1 DRGs (Figures 4C,D). The rightward shift in size distribution was most dramatic in L6/S1 DRGs, containing afferents innervating the urinary bladder and the distal colon (Nadelhaft and Booth, 1984).

### Capsaicin-sensitive afferents hypertrophied and upregulated the capsaicin receptor after spinal cord injury

While there was no change in the proportion of sensory neurons expressing TRPV1 after T3 SCI, this does not negate an intracellular upregulation of TRPV1. To investigate this possibility, we examined the intensity of TRPV1 expression in lumbosacral DRGs. Sections of L4/L5 and L6/S1 DRG from sham-injured controls and animals with T3 SCI were processed for TRPV1 immunohistochemistry and examined at the microscope by a blinded investigator, with all imaging parameters (exposure time, gain, and offset) set at constant levels across groups. With identical immunohistochemical processing and imaging, there was an obvious increase in intensity of TRPV1 expression in DRGs from animals with SCI (Figure 5). This was confirmed through blind quantification: when images were processed to generate size-intensity distributions of TRPV1-positive neuronal profiles, the intensity distribution in DRGs from animals with T3 SCI was right-shifted relative to sham-injured controls (Figures 5A,B). Akin to SCI-induced hypertrophy, the shift in TRPV1 signal intensity was most pronounced in the L6/S1 DRGs.

### Injury-induced hypertrophy was modest after low-thoracic spinal cord injury

The large majority of work in animal models of SCI examines injury-induced changes after low-thoracic SCI (Ramsey et al., 2010). For example, data describing SCI-induced changes in DRGs with bladder-projecting afferents (L6/S1), in the absence of changes in DRGs with somatic afferents (L4/L5), were derived from rats with low-thoracic SCI (Zvarova et al., 2005). We therefore examined the size distribution of TRPV1-positive afferents in lumbosacral DRGs from animals with T10 complete SCI (Figure 6). One month after T10 complete SCI, TRPV1-positive neurons did not exhibit hypertrophy in L4/L5 DRGs; size distribution of TRPV1-expressing neurons was similar between animals with T10 SCI and sham-injured controls (sham; Figure 6A). In the L6/S1 DRGs, there was a small but significant rightward shift in the size distribution of TRPV1-positive neurons after T10 SCI (Figure 6B). Interestingly, hypertrophic changes induced by low-thoracic SCI were much less dramatic than those triggered by T3 SCI (compare Figures 6B and 4D). This was surprising, given that both injuries induce hind limb paralysis and LUT dysfunction.

### Dramatic somatic hypertrophy in capsaicin-sensitive afferents was not reflected in plasticity of their central projections

Multiple studies have demonstrated that severe SCI triggers intraspinal sprouting of nociceptors (Krenz and Weaver, 1998; Weaver et al., 2001) and that sprouting of CGRP-expressing afferents in the dorsal horn is correlated with severity of AD (Krenz et al., 1999; Cameron et al., 2006). SCI also prompts a subset of DRG neurons, those expressing the pituitary adenylate cyclase activation peptide (PACAP), to expand their territory in the lumbosacral dorsal horn in segments containing visceral circuitry (L1, L2, L6, and S1; Zvarova et al., 2005). Since PACAP and CGRP partially co-localize with TRPV1 in DRG neurons (Moller et al., 1993), we measured the density of TRPV1-expressing terminals in the L4/L5 and L6/S1 dorsal horn, the central projections of afferents exhibiting the most pronounced hypertrophy after T3 SCI. Working from tiled mosaics of confocal z-stack projections (Figure 7A), we detected a slight but significant increase in density of TRPV1-positive terminals in the superficial laminae at two locations in the L4/L5 dorsal horn. Density of TRPV1-positive projections was increased superficially, in lamina I of the lateral dorsal horn and lamina II–III of the medial dorsal horn. There was no evidence of TRPV1-positive afferents sprouting into deeper laminae after SCI. There was also no difference in density of TRPV1-expressing afferents in the L6/S1 dorsal horn between sham-injured animals and animals with T3 SCI (Figure 7B). These results indicate that the CGRP- and PACAP-positive axons which were previously shown to sprout after SCI are not those which contain TRPV1.

We also examined density of TRPV1-positive projections to the DGC in the L6/S1 spinal cord, a region that receives input from visceral afferents, including those in the distal colon (Morgan et al., 1981; Hou et al., 2009). Densitometric analysis demonstrated that there was no effect of SCI on projections to the DGC (Figure 7B). Since we were working from cross-sections of spinal cord, we did not attempt quantitative measurements of TRPV1-positive projections to the lateral parasympathetic preganglionic nucleus (which is rostro-caudally periodic in the lumbosacral spinal cord; Morgan et al., 1981). There were no qualitatively apparent changes in the density of TRPV1-positive projections to parasympathetic preganglionic neurons after T3 SCI.

### Intrathecal capsaicin attenuated colo-rectal distension-induced autonomic dysreflexia

The dramatic effects of T3 SCI on TRPV1-expressing afferents in L6/S1 DRGs prompted us to examine their contribution to the development of AD (Figure 8). We administered 50 μg of capsaicin in a single intrathecal injection at the L4 spinal cord, 28 days after T3 SCI (“late Cap.”) or 48 h after T3 SCI (“early Cap.”). Since TRPV1-positive sensory neurons exhibit spontaneous activity *de novo* as early as 24 h following SCI (Bedi et al., 2010), we hypothesized that early capsaicin treatment might have particularly pronounced effects on development of AD. Vehicle injections (“Veh.”) were performed 28 days after SCI.

Consistent with previous findings, capsaicin injection produced a permanent degeneration of spinally projecting TRPV1-positive axons (Figure 8A; Yaksh et al., 1979). Efficacy of capsaicin was confirmed *via* densitometric measurements within the DGC (Figure 8A), which demonstrated a rapid and sustained depletion of TRPV1-positive projections. At 30 days after T3 SCI, carotid cannulae were implanted for beat-to-beat blood pressure measurements (Figure 8B). Capsaicin injection had no effects on blood pressure or HR at rest (Figure 8C). Animals that received capsaicin after T3 SCI exhibited much less severe AD in response to CRD (Figure 8C). There was no effect of differential timing of capsaicin injection following SCI: early and late capsaicin treatment produced equivalent reductions in CRD-induced hypertension and bradycardia. In both groups, the CRD-evoked change in SAP was reduced by approximately 50% relative to vehicle-treated animals, and CRD-induced bradycardia was dramatically attenuated.

## Discussion

These experiments demonstrate injury-induced hypertrophy in a specific subset of TRPV1-positive sensory neurons caudal to SCI. The response was most pronounced in lumbosacral DRGs and after high-thoracic SCI. Finally and notably, eliminating the central projections of TRPV1-expressing axons after T3 SCI *via* intrathecal capsaicin injection had pronounced mitigating effects on the severity of CRD-induced AD. Here we discuss the potential mechanisms of this SCI-provoked hypertrophy, its relationship to level of injury and the relevance for sensory-autonomic dysfunction following SCI.

### Spinal cord injury-induced hypertrophy was restricted to a subset of capsaicin-sensitive neurons

The selectivity of sensory neuron hypertrophy following SCI provides important clues about the underlying trophic mechanism. While the results demonstrate T3 SCI-induced hypertrophy of TRPV1-positive DRG neurons, it is clear that this is not universally true: that is, there must be a specific subset of TRPV1 neurons which responds to SCI by increasing in size. This assertion is based on the fact that a rightward shift in size-frequency distribution is detectable in the analysis of all (βIII-tubulin-labeled) neurons (Figure 1), but not for IB4/P2X_3_ or SP subpopulations (Figure 2), each of which partially co-localizes with TRPV1 (Tominaga et al., 1998). Thus, the TRPV1-expressing neurons of interest express neither standard peptidergic nor non-peptidergic nociceptor markers (Michael and Priestley, 1999); these would have been included in the analysis of βIII-tubulin-positive profiles (i.e., all DRG neurons), but omitted from those involving SP, P2X_3_, and IB4-positive cells. Thus, hypertrophy is largely or entirely restricted to a unique population of TRPV1-expressing neurons.

Analysis of neurons expressing GFRα3 (∼85% of which co-express TRPV1; Bennett et al., 2006) provides a further clue to the identity of this specific subset of nociceptors. The hypertrophic neurons are most likely sensitive to artemin, a GDNF family neurotrophic factor. Previous experiments have shown that peripheral over-expression of artemin in mouse keratinocytes not only leads to hypertrophy of DRG neurons, but also to upregulation of TRPV1 mRNA and increased capsaicin sensitivity (Elitt et al., 2006). Artemin is therefore a likely candidate for inducing hypertrophy following SCI. While there are no data describing expression of endogenous artemin after SCI, the pattern of injury-induced hypertrophy is suggestive.

### Spinal cord injury-induced hypertrophy was particularly dramatic in caudal ganglia, far distal to injury

The ganglia in which size changes occurred also give cause for speculation on the molecular underpinnings of this response to injury. The inflammatory response to SCI is prolonged and well-characterized (Alexander and Popovich, 2009). The character of the inflammatory milieu evolves over time and is accompanied by production of cytokines and trophic factors which could act on the ganglia attached to the cord to induce hypertrophy. However, injury-induced hypertrophy was absent or modest in T1 and T5 DRGs (respectively) which argues against a central role for a factor produced at the site of SCI.

Pronounced hypertrophy occurred far distal to the site of T3 SCI, in lumbosacral DRGs. Interestingly, TRPV1-positive somata were enlarged in DRGs supplying predominately somatic (L4/L5) and predominately visceral (L6/S1) peripheral targets. This scenario represents a departure from the phenotypic changes that are restricted to visceral afferents after low-thoracic injury (Kruse et al., 1995; Qiao and Vizzard, 2002). TRPV1-expressing sensory neurons innervate skin, epithelia (notably in the bladder), and both skeletal and smooth muscle in a variety of targets, including the colon and the bladder (Willis, 2007; Everaerts et al., 2008; Malin et al., 2011; Skryma et al., 2011; Yu et al., 2011). The structure and function of these target tissues are dramatically altered following SCI. Paralyzed skeletal muscle undergoes rapid and profound atrophy (Qin et al., 2010). In contrast, the smooth (detrusor) muscle of the bladder becomes hypertrophic over time following supraconal SCI, due to the combined effects of detrusor hyperactivity and detrusor-sphincter dyssynergia (Yoshimura, 1999). The bladder epithelium is also altered in chronic SCI (Smith et al., 2008). Less is known about remodeling of smooth muscle in the lower gastrointestinal (GI) tract after SCI. However, given the pronounced changes in lower GI function after SCI, including increased transit time that manifests clinically as constipation (Brading and Ramalingam, 2006), it seems reasonable to speculate that intrinsic smooth muscle of the distal colon also undergoes inactivity-induced remodeling following injury. The same logic applies to skin below the injury: while there are few data describing structural changes in skin, pressure ulcers are a common complication of SCI (Cardenas et al., 2004; Gelis et al., 2009). In view of the diverse changes in peripheral targets of TRPV1-positive lumbosacral DRG afferents after SCI, it is very likely that these afferents are exposed to different (or different amounts of) neurotrophic factors post-injury.

### Injury-induced hypertrophy was not accompanied by pronounced intraspinal sprouting

The pronounced somatic hypertrophy in TRPV1-expressing afferents was not accompanied by an equally dramatic expansion of their central terminals in the spinal dorsal horn. Previous findings indicate that low-thoracic SCI prompts an increase in TRPV1 mRNA, but a reduction in TRPV1 expression demonstrated immunohistochemically, at and around the site of injury (DomBourian et al., 2006). Distal to SCI, the only available data report that there is no change in spinal distribution of TRPV1-positive projections after low-thoracic injury (Cruz et al., 2008). We observed a relatively minor increase in density that was restricted to the superficial laminae of the L4/L5 dorsal horn after T3 SCI. While somatic hypertrophy and axonal sprouting do not necessarily occur together, most trophic factors are capable of eliciting both. For example, in bladder afferents caudal to low-thoracic SCI, bladder-, and/or spinal cord-derived NGF is thought to mediate somatic hypertrophy (Yoshimura, 1999; Seki et al., 2002; Yoshimura et al., 2006), and intraspinal NGF also contributes to SCI-induced sprouting of peptidergic nociceptors in the dorsal horn (Christensen and Hulsebosch, 1997; Krenz et al., 1999; Cameron et al., 2006). In contrast to the distribution of CGRP-positive terminals in the dorsal horn caudal to high-thoracic SCI (Ondarza et al., 2003), TRPV1-positive afferents did not invade deeper laminae of the dorsal horn. In these experiments, we demonstrate something quite different after SCI: afferents that are constitutively present in the spinal dorsal horn undergo dramatic hypertrophy and receptor upregulation at the level of their soma (i.e., in the periphery). While the majority of data describe dramatic changes within the spinal cord caudal to high-thoracic SCI, creating the potential for altered connectivity within central sensory-sympathetic circuits, we demonstrate pronounced effects in the periphery. The potential for pathological sensory-sympathetic interactions in the periphery exists, and may also contribute to AD (Ramer et al., 2006). The absence of robust sprouting of TRPV1 axons in the spinal cord suggests that the stimulus is also peripheral, possibly in the DRG itself. An intra-ganglionic source of artemin, for example, might be satellite cells in the DRG: artemin is expressed in Schwann cells, which are phenotypically similar to satellite cells, and is upregulated in Schwann cells after peripheral nerve injury (Baloh et al., 1998).

### Injury-induced hypertrophy was more pronounced after high-thoracic than low-thoracic spinal cord injury

The differential effects of complete SCI at T3 and T10 are interesting, given the similarities in many aspects of functional outcome. For example, T3 and T10 SCI induce hind limb paralysis and bladder dysfunction that is grossly similar: the bladder is initially areflexic and requires manual emptying until reflexive micturition is restored. The issue of LUT function is certainly relevant, since TRPV1-positive sensory neurons are critically involved in physiological bladder function (Araki, 2011) and appear to contribute to bladder dysfunction after SCI (Cruz et al., 2008). TRPV1-expressing afferents also mediate a number of pathological phenomena in the LUT, including bladder pain and overactive bladder (Eid, 2011; Kissin and Szallasi, 2011); recent findings suggest that these afferents also participate in pelvic organ cross-sensitization (Asfaw et al., 2011). However, SCI-induced changes were not restricted to bladder afferents after T3 SCI, since hypertrophy was also apparent in L4/L5 DRGs. One explanation might be that signals from the hypertrophic bladder contribute to injury-induced hypertrophy, but the predominant trigger is present in high-, but not low-thoracic SCI.

One notable difference in high- versus low-thoracic SCI that has been identified lies in the immune response to injury. In mice, immune suppression induced by SCI is level-dependent, such that mice with T3 SCI exhibit impaired antibody synthesis and elevated splenic norepinephrine, neither of which develop in mice with T9 injury (Lucin et al., 2007). If level-dependent immune suppression also occurs in rats with SCI, the inflammatory response in the DRG may also vary with level of injury, influenced by systemic activity of the immune system. The limited data that are available describe immune cell infiltration in DRGs caudal to T8 SCI (McKay and McLachlan, 2004): in this study, intra-ganglionic immune cell density was highest in DRGs closer to the lesion site (i.e., greater in T12 DRGs than in L6 DRGs). This pattern does not seem to fit with our results, but may be different after T3 SCI; alternatively, other factors modifying the environment of the DRG (such as satellite cells activation) may not vary in step with the local immune response.

The most dramatic difference in the functional outcomes of T3 and T10 SCI is the development of AD after the former, but not the latter injury. In AD, sensory stimulation evokes sympathetic contractions of vascular smooth muscle. It is not insignificant that artemin is developmentally expressed in vascular smooth muscle where it acts as a guidance cue for sympathetic (and probably also sensory) axons (Honma et al., 2002). In essential hypertension, chronic constriction of the blood vessels induces maladaptive remodeling of the vasculature (Rizzoni et al., 2007, 2009; Rehman and Schiffrin, 2010). Remodeling resulting in an increase in media-lumen ratio can occur *via* different mechanisms, including rearrangement of the same wall material around a narrowed lumen (eutrophic remodeling) or vascular smooth muscle cell growth (hypertrophic remodeling; Intengan and Schiffrin, 2001). While less is known about the effects of intermittent or episodic hypertension on vascular structure, one recent study indicates that carotid intima-media thickness is increased in individuals with SCI (Matos-Souza et al., 2009). The possibility of artemin upregulation in vascular smooth muscle has yet to be explored, but may contribute to the more pronounced hypertrophy of TRPV1/GFRα3 neurons following T3 SCI compared to injury at T10. TRPV1-positive afferents have collateral branches that supply blood vessels, particularly arterioles, in the submucosa of the gastrointestinal (GI) tract (Holzer, 2006). The vasculature of the GI tract is part of the splanchnic bed that is critically involved in blood pressure control, including the development of AD (Lujan et al., 2010): these arteries are known to be altered after high-thoracic SCI (Brock et al., 2006; Alan et al., 2010).

### Capsaicin-sensitive afferents contributed substantially to colo-rectal distension-induced autonomic dysreflexia

Selective elimination of TRPV1-expressing afferents in the spinal dorsal horn dramatically reduced the severity of AD. Peripheral projections of TRPV1-positive afferents in the DRGs innervating the rectum and distal colon are found in smooth muscle and the mucosa and are mechanosensitive and/or chemosensitive (Lynn and Blackshaw, 1999; Berthoud et al., 2001; Ward et al., 2003). Neurons in the capsaicin-sensitive, mechanosensitive subset are known to respond to CRD (Brierley et al., 2005). Intrathecal capsaicin injected at L4 eliminated the central projections of TRPV1-positive spinal colonic afferents, which constitute approximately 50% of the lumbosacral colonic DRG neurons (Brierley et al., 2005). This is reflected in our data, demonstrating that CRD following intrathecal capsaicin injection still activates a subset of mechanosensitive afferents to elicit AD, although AD is dramatically reduced in severity (Figure 8).

We hypothesized that early elimination of TRPV1-expressing afferents would have even more pronounced effects on AD (than late capsaicin, administered at 28 days post-SCI). This premise was based in part on recent findings demonstrating spontaneous activity arising in the soma develops in DRGs after SCI (Bedi et al., 2010). There are several striking similarities between the patterns of *de novo* spontaneous activity and hypertrophy that emerge following SCI. Both phenomena develop caudal, but not rostral to SCI, and are most pronounced in distal DRGs, remote from the site of injury. Both occur in nociceptors, and most intriguingly, a high percentage of the afferents that exhibited spontaneous activity after T10 SCI were capsaicin-sensitive (Bedi et al., 2010). Cumulatively, these findings suggest that SCI has specific effects on TRPV1-expressing primary afferents. In bladder afferents, SCI-induced somatic hypertrophy is accompanied by increased excitability, including reduced thresholds for activation (Yoshimura, 1999). If hypertrophy is an anatomical surrogate for spontaneous activity after SCI, injury-induced ongoing activity in TRPV1-positive neurons might be more even more pronounced and/or prevalent after T3 SCI.

However, early and late capsaicin treatment had equivalent effects on AD (Figure 8B). From these findings, we cannot reliably determine whether TRPV1-positive afferents only instigate CRD-evoked AD, or both instigate AD and contribute to its development over time following SCI. A reversible TRPV1-block might distinguish between these two possibilities. Given the evidence for spontaneous activity in capsaicin-sensitive afferents caudal to SCI, this is a future direction with important clinical implications for AD, and potentially for pain.

## Conclusion

Previous work has identified numerous mechanisms that might contribute to induction and progression of AD, and the list of putative mechanisms includes injury-induced changes in the vasculature and multiple components of the spinal sensory-sympathetic circuitry caudal to SCI (Krenz and Weaver, 1998; Krassioukov et al., 1999; Krenz et al., 1999; Brock et al., 2006; McLachlan and Brock, 2006). In terms of sensory plasticity, prior findings demonstrate that severity of AD is closely correlated to the extent of intraspinal nociceptor sprouting (Cameron et al., 2006). However, this is the first study to demonstrate AD mediated by a specific subset of afferents that exhibit pronounced somatic, but only slight central, injury-induced plasticity. Given the array of pronounced changes in peripheral targets of sensory neurons after SCI, it is not surprising that they respond to injury. Plasticity occurring outside the CNS may represent a new and more accessible target for limiting sensory-autonomic dysfunction following SCI.

## Conflict of Interest Statement

The authors declare that the research was conducted in the absence of any commercial or financial relationships that could be construed as a potential conflict of interest.
